# Investigating associated factors of primary and specialist health care utilization among people with selected nationalities: results of a multilingual survey in two German federal states

**DOI:** 10.1186/s12913-022-08419-y

**Published:** 2022-08-17

**Authors:** Anne-Kathrin M. Loer, Carmen Koschollek, Claudia Hövener

**Affiliations:** Department of Epidemiology and Health Monitoring, Robert Koch Institute, General-Pape-Str. 62-66, 12101 Berlin, Germany

**Keywords:** Migration history, Utilization of health services, General and specialist medical care, Access to care

## Abstract

**Background:**

Approximately every fourth person in Germany has a migration background. Health research on the use of primary and specialist health care in this group is still scarce. Few studies have suggested a difference in the use of primary and specialist health care among people with a migration background. Potential resources and barriers to health care access should be investigated as they are critical to health equity. This study investigates associated sociodemographic, migration-sensitive, and health-related factors of primary and specialist health care utilization among people with a migration background as defined by nationality.

**Methods:**

Analyses are based on data from a feasibility study of the project “Improving Health Monitoring in Migrant Populations” (IMIRA), conducted by the Robert Koch Institute. The sample (*n* = 1055) included persons with Croatian, Polish, Romanian, Syrian, and Turkish nationalities living in the federal states of Berlin and Brandenburg, Germany. Descriptive and bivariate analyses as well as multiple binary logistic regression analyses were carried out to assess sociodemographic (sex, age, socioeconomic position), health-related (self-rated health), and migration-sensitive factors (duration of residence in Germany, residence status, German language proficiency) associated with the use of primary and specialist health care services in the past 12 months.

**Results:**

Of the total study population, 79.62% visited a general practitioner and 59.53% a specialized physician in the past 12 months. Participants who were female sex, aged 65 and older, and with moderate/poor/very poor self-rated health had higher odds of visiting a general practitioner and a specialized physician, with the strongest impact from self-rated health. After controlling for sociodemographic and health-related factors, duration of residence in Germany and residence status were associated with primary but not with specialist health care utilization.

**Conclusions:**

Our results suggest that migration-sensitive characteristics, such as duration of residence, should be considered in a differentiated manner in health services research to gain detailed insights into health care utilization and its potential barriers among the heterogenous group of people with a migration background. Further research needs to be done to evaluate how to get people into contact with a general practitioner.

## Background

### Introduction

The proportion of people with a statistically defined migration background in the German general population has been rising for years [1] and was at 26.7% in 2020. The statistical category of “people with migration background” refers to people who were born or had at least one parent born with a nationality other than German, and hereby subsumes people with very diverse backgrounds [2]; for example, so called “guest workers” from Southern Europe and Turkey who were recruited to Germany in the 1950s and 1960s as a workforce to bridge the labor shortage and who stayed – along with their descendants – as well as refugees who have recently arrived in Germany [3, 4].

Ambulatory health care in Germany plays a key role in the health care of the population because prevention, rehabilitation, and health promotion are primarily embedded in this sector [5]. This type of care is mainly provided by general practitioners and specialized physicians who are the first point of contact for patients with health-related problems. They perform most diagnoses, treat patients, and refer them to the hospital if needed [6, 7]. Primary health care is mainly provided by general practitioners and especially offers family health care services. Specialist health care is provided by specialists focusing on a specific medical field, such as gynecology or dermatology [8]. Evidence about the use of ambulatory health care and its associated factors among people with a migration background is important as health care utilization reflects health status [9, 10]; a lower utilization of health care services can have negative effects on health [9, 11] whereby poorer health conditions can result in higher utilization of health services [12, 13]. Data about the use of ambulatory health care by people with a migration background can be used to develop and implement well-founded measures to improve access to services [5, 14]. However, data about the use of primary and specialist health care services, and especially its associated factors, among people with a migration background in Germany are still scarce [5].

A few studies have suggested a difference in the use of primary and specialist health care among people with and without a migration background in Germany [15–18]. People with a migration background use preventive and rehabilitative measures to a lesser extent [15, 19–22], for example, clinical examinations for early detection of various diseases [17, 19, 23, 24]. Graetz et al. conducted a systematic review of the use of health care services by migrants in Europe and found that studies comparing the use of general practitioners’ services among migrants and non-migrants in various countries were inconsistent [25]. With respect to specialist health care, several of the studies described a generally lower utilization among migrants [25]. In contrast, visits to emergency departments by people with a migration background were observed to occur more frequently in Germany [26, 27], which is in line with findings in other European countries [28]. The higher utilization of emergency departments hints to the existence of barriers to access of ambulatory health care services [29].

Generally, the use of primary and specialist health care is associated with sociodemographic and health-related factors; for example, a very good/good self-rated state of health, a high socioeconomic position, male sex and younger age are associated with less frequent utilization or a lower frequency of contact with primary and specialist health care services [14, 30–34]. But there might be other barriers in accessing health services, especially for people with a migration background. Specific migration-sensitive factors can affect peoples’ use of health services: study results have suggested that region of origin, reason for migration, migration generation, residence status, knowledge of the language of the country of residence, duration of residence, and discrimination and racism within the health care sector are potential indicators of differences in health care use among people with a migration background compared to people without a migration background [16, 17, 26, 30, 35–41].Two studies have shown that migration-sensitive factors also lead to differences in utilization within the group of people with a migration background: Glaesmer et al. examined the health care utilization among first and second generation migrants and native-born Germans and found a more frequent use of general practitioners but a less frequent use of specialists among first generation migrants compared to second generation migrants [16]. Borde et al. observed that people with a Turkish migration background more often used a general practitioner compared to people with a non-Turkish migration background [26].

Besides these study results, evidence about differences in utilization of ambulatory health care services among the heterogeneous group of people with a migration background and its associated factors is lacking.

Therefore, the present study investigated the following research question: After controlling for sociodemographic and health-related factors, are migration-sensitive factors (duration of residence in Germany, residence status, and German language proficiency) associated with the utilization of primary and specialist health care services among people with selected nationalities in two German federal states, Berlin and Brandenburg?

## Methods

### Data collection

The feasibility study was one of eight sub-projects of the project “Improving Health Monitoring in Migrant Populations (IMIRA)”, conducted by the Robert Koch Institute (RKI) [42]. The feasibility study tested various interventions and modes of administration with regard to their applicability in increasing response rates in interview surveys among people with selected nationalities. The target population consisted of people aged 18 and older with Croatian, Polish, Romanian, Syrian, or Turkish nationality. A two-stage stratified cluster sampling strategy was applied. Seven primary sampling units (PSU) within two German federal states were selected: Berlin (five districts: Mitte, Neukölln, Charlottenburg-Wilmersdorf, Friedrichshain-Kreuzberg, and Tempelhof-Schöneberg) was selected to represent urban regions and Brandenburg (two cities with at least 33,000 inhabitants: Cottbus and Fürstenwalde/Spree) to represent less urban regions. Within these sample points, a pre-defined number of addresses (*n* = 9068 in total) were randomly drawn from local population registers at resident registration offices [43]. Moving into a residence in Germany, both Germans and Non-Germans have to register within 2 weeks at the resident registration offices of their municipality [44]. Data was collected from January to May 2018 using a slightly modified “European Health Interview Survey” (EHIS) [45]. Items to capture migration status and other migration-sensitive aspects were added [46]. Participants were sequentially able to take part via online, telephone, or personal interview in the languages of Arabic, Croatian, German, Polish, Romanian, or Turkish. The overall response rate was 15.9% [43]. The study concept, design, and methodology are described in more detail elsewhere [43, 46].

### Measures

#### Outcome measures “primary and specialist health care utilization in the past twelve months”

The outcome measure indicators were (1) “at least one-time utilization of *primary health care* during the past 12 months” and (2) “at least one-time utilization of *specialist health care* during the past 12 months.” Data on these indicators was collected using the following questions: (1) “When was the last time you consulted a GP (general practitioner) on your own behalf?” and (2) “When was the last time you consulted a medical or surgical specialist on your own behalf for advice, examination, or treatment?” The response options were: “Less than 12 months ago,” “12 months ago or longer,” and “Never.” The question on the utilization of specialist health care was only asked if the participant answered that they had visited a general practitioner less than 12 months ago. Two binary variables for (1) primary and (2) specialist health care utilization in the past 12 months were created (yes = “Less than 12 months ago”; no = “12 months ago or longer” or “Never”). Participants who had not visited a general practitioner during the past 12 months and therefore had a missing value in the variable specialist health care utilization were assigned to the answer category “No.”

#### Sociodemographic factors

Information on gender and age is based on the data supplied by the residents’ registration offices. Age was calculated using the year of birth and grouped into four categories (18 to 29 years; 30 to 44 years**;** 45 to 64 years**;** ≥ 65 years). An index for the socioeconomic position (SEP) was calculated, ranging from 3 to 21 points, based on information on education, income, and occupation. The index classified participants into three groups (low/ middle/high SEP) [47]; within this classification, the lower 20% of the index within the sample usually corresponded to the low SEP group and the upper 20% to the high SEP group [48]. The threshold values of the most recent representative survey among the general population in Germany (“German Health Update 2014/15” GEDA) conducted by the RKI were used to compare the socioeconomic position within the sample with that of the general population.

#### Health-related factor

Study participants were asked to rate their health by answering the following question: “How is your health in general?” Responses were dichotomized (“very good/good” vs. “moderate/poor/very poor”).

#### Migration-sensitive factors

The term “migration-sensitive” refers to the term “migration-specific” used by Razum & Spallek (2014) [49] and includes both immigrants and their descendants. Duration of residence in Germany, residence status and self-rated German language proficiency were included as migration-sensitive factors. Duration of residence was determined by a person’s country of birth and the year he or she moved to Germany if the country of birth was not Germany (duration of residence: since birth, < 2 years**;** 2 years to 10 years**;** > 10 years). Answering options related to residence status were grouped into the categories “German nationality,” “permanent residence status” (settlement permit, i.e., a permanent residence permit in Germany, or an EU long-term residence permit) and “temporary residence status” (a temporary residence permit, EU Blue Card, permission to reside, or temporary suspension of deportation). Responses regarding self-assessed German language proficiency were combined into two categories (“Mother tongue/very good/good” vs. “Moderate/poor/very poor”).

### Statistical analyses

All participants with at least one missing value in one of the aforementioned variables were excluded listwise from analyses. Absolute frequencies and percentages were calculated to describe sample characteristics. A Kruskal-Wallis H test was used to test for differences between selected nationalities and sociodemographic, health-related and migration-sensitive factors.

Associations between potentially related factors and at least one-time utilization of (1) primary or (2) specialist health care during the past 12 months were explored by using Pearson’s chi-squared tests using a significance level of *p* ≤ 0.05 for the indicator variables. Multiple binary logistic regression models estimating odds ratios (OR) with 95% confidence intervals (95%-CI) were tested in a block-wise modeling approach to model relations between potentially related factors and at least one-time utilization of (1) primary or (2) specialist health care during the past 12 months. In a first model, associations between sociodemographic factors and the utilization of health care services were examined. In a second model, the association between self-rated health and utilization of health care services was assessed while controlling for sociodemographic factors. Multicollinearity was found between residence status and duration of residence in Germany. As variables that are highly collinear can cause problems (the statistical significance of an independent variable is undermined) [50], two distinct models were calculated in a third step (Model 3a including duration of residence in Germany, Model 3b including residence status). In this way, the impact of either residence status or duration of residence in Germany on the utilization of health care services was explored while controlling for sociodemographic and health-related factors. The variable German language proficiency was excluded from multiple binary logistic regression analyses due to interaction effects with both of the other migration-sensitive factors (residence status and duration of residence), tested by an adjusted Wald test, and due to a lack of associations with health care utilization in bivariate analyses and an almost entire lack of associations in further analyses (significance in only one in four models, not shown). Data analyses were performed using Stata version 15.1.

## Results

### Description of the study population

Overall, 1190 people took part in the survey; the listwise exclusion of cases with at least one missing value in one of the described variables resulted in a final sample size of 1055 participants. Within the sample, 51.47% of the participants were female. The median age was 45.4 years. Approximately half of the participants (45,50%) were classified as having a low socioeconomic position. Most of the participants had migrated themselves. Approximately one-third of the study population rated their health as moderate, poor, or very poor (Table 1).

**Table 1:** Characteristics of the study population stratified by nationality (*n* = 1055)

		Croatian nationality (*n* = 157)	Polish nationality (*n* = 197)	Rumanian nationality (*n* = 87)	Syrian nationality (*n* = 416)	Turkish nationality (*n* = 198)	Total study population (*n* = 1.055)
*Sociodemographic* *Factors*	Sex^a^						
	Male	45.86% (*n* = 72)	46.70% (*n* = 92)	39.08% (*n* = 34)	54.57% (*n* = 227)	43.94% (*n* = 87)	48.53% (*n* = 512)
	Female	54.14% (*n* = 85)	53.30% (*n* = 105)	60.92% (*n* = 53)	45.43% (*n* = 189)	56.06% (*n* = 111)	51.47% (*n* = 543)
	Age (Mean/SD)^a^	47.2 (17.4)	49.4 (15.2)	44.9 (16.1)	42.00 (15.8)	47.1 (17.6)	45.4 (16.6)
	18 to 29 years	19.11% (*n* = 30)	9.14% (*n* = 18)	18.39% (*n* = 16)	27.64% (*n* = 115)	20.20% (*n* = 40)	20.76% (*n* = 219)
	30 to 44 years	28.66% (*n* = 45)	26.90% (*n* = 53)	33.33% (*n* = 29)	29.09% (*n* = 121)	26.26% (*n* = 52)	28.44% (*n* = 300)
	45 to 64 years	22.93% (*n* = 36)	41.12% (*n* = 81)	29.89% (*n* = 26)	31.01% (*n* = 129)	30.30% (*n* = 60)	31.47% (*n* = 332)
	≥ 65 years	29.30% (*n* = 46)	22.84% (*n* = 45)	18.39% (*n* = 16)	12.26% (*n* = 51)	23.23% (*n* = 46)	19.34% (*n* = 204)
	Socioeconomic position^a^						
	Low	26.11% (*n* = 41)	23.86% (*n* = 47)	37.93% (*n* = 33)	57.45% (*n* = 239)	60.61% (*n* = 120)	45.50% (*n* = 480)
	Middle	56.69% (*n* = 89)	52.79% (*n* = 104)	37.93% (*n* = 33)	35.58% (*n* = 148)	31.82% (*n* = 63)	41.42% (*n* = 437)
	High	17.20% (*n* = 27)	23.35% (*n* = 46)	24.14% (*n* = 21)	06.97% (*n* = 29)	07.58% (*n* = 15)	13.08% (*n* = 138)
*Health-related factor*	Self-rated health^a^						
	Very good/good	75.16% (*n* = 118)	76.14% (*n* = 150)	78.16% (*n* = 68)	70.19% (*n* = 292)	61.62% (*n* = 122)	71.09% (*n* = 750)
	Moderate/poor/very poor	24.84% (*n* = 39)	23.86% (n = 47)	21.84% (*n* = 19)	29.81% (*n* = 124)	38.38% (*n* = 76)	28.91% (*n* = 305)
*Migration-sensitive factors*	Duration of residence in Germany^a^						
	Since birth	26.75% (*n* = 42)	07.11% (*n* = 14)	01.15% (*n* = 1)	04.09% (*n* = 17)	34.34% (*n* = 68)	13.46% (*n* = 142)
	< 2 years	07.01% (*n* = 11)	04.06% (*n* = 8)	09.20% (*n* = 8)	26.44% (*n* = 110)	01.01% (*n* = 2)	13.18% (*n* = 139)
	2 years to 10 years	12.10% (*n* = 19)	21.32% (*n* = 42)	49.43% (*n* = 43)	53.61% (*n* = 223)	04.55% (*n* = 9)	31.85% (*n* = 336)
	> 10 years	54.14% (*n* = 85)	67.51% (*n* = 133)	40.23% (*n* = 35)	15.87% (*n* = 66)	60.10% (*n* = 119)	41.52% (*n* = 438)
	Residence status^a^						
	German nationality	17.83% (*n* = 28)	49.75% (*n* = 98)	39.08% (*n* = 34)	14.66% (*n* = 61)	25.76% (*n* = 51)	25.78% (*n* = 272)
	Permanent residence status	80.25% (*n* = 126)	49.24% (*n* = 97)	60.92% (*n* = 53)	02.16% (*n* = 9)	60.61% (*n* = 120)	38.39% (*n* = 405)
	Temporary residence status	01.91% (*n* = 3)	01.02% (*n* = 2)	00.00% (*n* = 0)	83.17% (*n* = 346)	13.64% (*n* = 27)	35.83% (*n* = 378)
	German language proficiency^a^						
	Mother tongue/very good/good	78.34% (*n* = 123)	73.10% (*n* = 144)	64.37% (*n* = 56)	31.01% (*n* = 129)	55.05% (*n* = 109)	53.18% (*n* = 561)
	Moderate/poor/very poor	21.66% (*n* = 34)	26.90% (*n* = 53)	35.63% (*n* = 31)	68.99% (*n* = 287)	44.95% (*n* = 89)	46.82% (*n* = 494)

^a^ Results of Kruskal-Wallis H Test: indicates for *p* ≤ 0.05

### Primary health care utilization in the past 12 months

More than three in four participants reported having visited a general practitioner in the past 12 months. Sex, age, duration of residence in Germany, residence status, and self-rated health were associated with visiting a general practitioner (Table 2). No associations were found regarding socioeconomic position and German language proficiency.

**Table 2:** Factors associated with the utilization of primary health care: Results of bivariate analyses (*n* = 1055)

		n	% of study population with utilization of primary health care services in the past twelve months (95%-CI)	*p*-value^a^
	Total	840	79.62 (77.06–82.01)	
*Sociodemographic factors*	Sex			**< 0.001**
	Male	384	75.00 (71.06–78.57)	
	Female	456	83.98 (80.64–86.83)	
	Age			**< 0.001**
	18 to 29 years	158	72.15 (65.82–77.70)	
	30 to 44 years	226	75.33 (70.12–79.89)	
	45 to 64 years	273	82.23 (77.73–85.99)	
	≥ 65 years	183	89.71 (84.71–93.20)	
	Socioeconomic position			0.27
	Low	376	78.33 (74.41–81.80)	
	Middle	358	81.92 (78.02–85.26)	
	High	106	76.81 (69.01–83.13)	
*Health-related factor*	Self-rated health			**< 0.001**
	Very good/good	569	75.87 (72.67–78.80)	
	Moderate/poor/very poor	271	88.85 (84.79–91.93)	
*Migration-sensitive factors*	Duration of residence in Germany			**< 0.001**
	Since birth	116	81.69 (74.43–87.24)	
	< 2 years	91	65.47 (57.16–72.93)	
	2 years to 10 years	255	75.89 (71.02–80.18)	
	> 10 years	378	86.30 (82.74–89.22)	
	Residence status			**< 0.001**
	German nationality	226	83.09 (78.14–87.10)	
	Permanent residence status	345	85.19 (81.37–88.33)	
	Temporary residence status	269	71.16 (66.38–75.52)	
	German language proficiency			0.68
	Mother tongue/very good/good	444	79.14 (75.58–82.31)	
	Moderate/poor/very poor	396	80.16 (76.40–83.45)	

^a^ Conducted by Pearson’s chi-squared tests. Boldface indicates *p* < 0.05. *Abbreviation: CI* confidence interval

In model 1, participants with female sex and aged 45 years and over were more likely to have visited a general practitioner in the past 12 months.

After including self-rated health in model 2, the association for the age category 45 to 64 years could no longer be observed. Participants with a moderate/poor/very poor self-rated health had higher odds of having visited a general practitioner.

Residing in Germany less than 2 years (Model 3a) and having a temporary residence status (Model 3b) decreased the odds of having visited a general practitioner.

No association between socioeconomic position and visiting a general practitioner was observed in any of the models (Table 3).

**Table 3:** Factors associated with the utilization of primary health care: Results of multiple binary logistic regression analyses (*n* = 1055)

	Indicator Variable	Model 1 OR (95% - CI)	Model 2 OR (95% - CI)	Model 3a OR (95% - CI)	Model 3b OR (95% - CI)
*Sociodemographic* *factors*	Sex				
	Male	Ref.	Ref.	Ref.	Ref.
	Female	**1.86 (1.36–2.54)**	**1.83 (1.34–2.49)**	**1.87 (1.36–2.58)**	**1.76 (1.29–2.42)**
	Age				
	18 to 29 years	Ref.	Ref.	Ref.	Ref.
	30 to 44 years	1.15 (0.77–1.72)	1.10 (0.73–1.66)	1.07 (0.70–1.63)	1.06 (0.70–1.60)
	45 to 64 years	**1.80 (1.19–2.72)**	1.50 (0.98–2.29)	1.38 (0.86–2.19)	1.37 (0.89–2.10)
	≥ 65 years	**3.45 (2.00–5.95)**	**2.72 (1.56–4.77)**	**2.26 (1.20–4.27)**	**2.19 (1.23–3.89)**
	Socioeconomic position				
	Low	Ref.	Ref.	Ref.	Ref.
	Middle	1.22 (0.87–1.70)	1.34 (0.95–1.89)	1.23 (0.88–1.71)	1.17 (0.82–1.67)
	High	1.01 (0.63–1.61)	1.20 (0.75–1.94)	1.13 (0.70–1.82)	0.93 (0.56–1.55)
*Health-related factor*	Self-rated health				
	Very good/good		Ref.	Ref.	Ref.
	Moderate/poor/very poor		**2.13 (1.40–3.25)**	**2.10 (1.37–3.20)**	**2.09 (1.38–3.16)**
*Migration-sensitive factors*	Duration of residence in				
	Germany				
	Since birth			Ref.	
	< 2 years			**0.36 (0.21–0.64)**	
	2 years to 10 years			0.64 (0.38–1.06)	
	> 10 years			0.84 (0.47–1.48)	
	Residence status				
	German nationality				Ref.
	Permanent residence status				1.21 (0.79–1.86)
	Temporary residence status				**0.57 (0.37–0.88)**

Boldface indicates *p* ≤ 0.05. *Abbreviations: CI* confidence interval, *OR* odds ratio, *Ref*. reference category

### Specialist health care utilization in the past 12 months

Approximately six in ten participants reported having used the services of a specialized physician in the past 12 months. Visiting a specialized physician was associated with sex, age, self-rated health, and duration of residence in Germany but not with socioeconomic position, residence status, and German language proficiency (Table 4).

**Table 4:** Factors associated with the utilization of specialist health care: Results of bivariate analyses (*n* = 1055)

		n	% of study population with utilization of specialist health care services in the past twelve months (95%-CI)	*p*-value^a^
	Total	628	59.53 (56.94–62.51)	
*Sociodemographic* *factors*	Sex			**0.004**
	Male	282	55.08 (50.73–59.35)	
	Female	346	63.72 (59.58–67.67)	
	Age			**< 0.001**
	18 to 29 years	144	52.05 (45.42–58.62)	
	30 to 44 years	160	53.33 (47.65–58.93)	
	45 to 64 years	206	62.05 (56.69–67.13)	
	≥ 65 years	148	72.55 (65.70–78.25)	
	Socioeconomic position			0.68
	Low	291	60.62 (56.17–64.91)	
	Middle	259	59.27 (54.58–63.79)	
	High	78	56.52 (48.11–65.58)	
*Health-related factor*	Self-rated health			**< 0.001**
	Very good/good	397	52.93 (49.35–56.94)	
	Moderate/poor very poor	231	75.74 (70.60–80.23)	
*Migration-sensitive factors*	Duration of residence in Germany			**0.001**
	Since birth	76	53.52 (45.26–61.60)	
	< 2 years	73	52.52 (44.18–60.71)	
	2 years to 10 years	188	55.96 (50.58–61.19)	
	> 10 years	291	66.44 (61.87–70.72)	
	Residence status			0.08
	German nationality	161	59.19 (53.23–64.89)	
	Permanent residence status	257	63.46 (58.64–68.02)	
	Temporary residence status	210	55.56 (50.49–60.50)	
	German language proficiency			0.62
	Mother tongue/very good/good	330	58.82 (54.69–62.84)	
	Moderate/poor/very poor	298	60.32 (55.93–64.56)	

^a^ Conducted by Pearson’s chi-squared tests. Boldface indicates *p* < 0.05. *Abbreviations: CI* confidence interval

In model 1, being female or aged 45 years or over increased the odds of having visited a specialized physician in the past 12 months.

After the inclusion of self-rated health in Model 2, the age category 45 to 64 years was no longer associated with visit to a specialized physician. Participants with a moderate/poor/very poor self-rated health were more likely to have visited a specialized physician.

Overall, socioeconomic position, duration of residence in Germany, and residence status were not associated with specialist health care utilization in the past 12 months (Table 5).

**Table 5:** Factors associated with the utilization of specialist health care: Results of multiple binary logistic regression analyses (*n* = 1055)

	Indicator Variable	Model 1 OR (95% - CI)	Model 2 OR (95% - CI)	Model 3a OR (95% - CI)	Model 3b OR (95% - CI)
*Sociodemographic factors*	Sex				
	Male	Ref.	Ref.	Ref.	Ref.
	Female	**1.49 (1.16–1.92)**	**1.46 (1.13–1.89)**	**1.46 (1.13–1.89)**	**1.44 (1.12–1.87)**
	Age				
	18 to 29 years	Ref.	Ref.	Ref.	Ref.
	30 to 44 years	1.05 (0.74–1.50)	0.99 (0.69–1.42)	0.96 (0.67–1.39)	0.98 (0.68–1.40)
	45 to 64 years	**1.54 (1.08–2.18)**	1.22 (0.85–1.75)	1.14 (0.77–1.69)	1.20 (0.83–1.72)
	≥ 65 years	**2.54 (1.68–3.83)**	**1.89 (1.23–2.90)**	**1.69 (1.04–2.76)**	**1.81 (1.17–2.81)**
	Socioeconomic position				
	Low	Ref.	Ref.	Ref.	Ref.
	Middle	0.92 (0.70–1.21)	1.04 (0.79–1.38)	1.03 (0.78–1.36)	1.02 (0.76–1.36)
	High	0.91 (0.61–1.36)	1.17 (0.78–1.76)	1.14 (0.76–1.72)	1.14 (0.74–1.74)
*Health-related factor*	Self-rated health				
	Very good/good		Ref.	Ref.	Ref.
	Moderate/poor/very poor		**2.46 (1.79–3.39)**	**2.44 (1.77–3.36)**	**2.47 (1.79–3.40)**
Migration-sensitive factors	Duration of residence in				
	Germany				
	Since birth			Ref.	
	< 2 years			0.87 (0.53–1.40)	
	2 years to 10 years			0.99 (0.66–1.49)	
	> 10 years			1.12 (0.72–1.74)	
	Residence status				
	German nationality				Ref.
	Permanent residence status				1.17 (0.84–1.63)
	Temporary residence status				0.95 (0.66–1.35)

Boldface indicates *p* ≤ 0.05. *Abbreviations: CI* confidence interval, *OR* odds ratio, *Ref*. reference category

## Discussion

Our study aimed to examine the sociodemographic, migration-sensitive and health-related factors associated with (1) primary and (2) specialist health care utilization among people with selected nationalities in two German federal states, Berlin and Brandenburg. More than three in four participants visited a general practitioner and more than half consulted a specialized physician in the past 12 months. Participants with female sex, aged ≥65 years and poor self-rated health were more likely to have visited a general practitioner and a specialized physician, with self-rated health showing the strongest effect. Duration of residence in Germany and residence status were associated with primary but not specialist health care utilization, even after controlling for sociodemographic and health-related factors.

### Primary and specialist health care utilization

Approximately 80% of our sample used the services of general practitioners and about 60% used the services of specialized physicians in the past 12 months. Based on a nation-wide representative sample of the Robert Koch Institute’s German Health Interview and Examination Survey for Adults (DEGS1, 2008–2011), two studies examined prevalence rates for health care utilization [14, 34]. One of the studies found that 79.4% of the study population – the general population in Germany – had visited a general practitioner at least once in the past 12 months [14]. The other study – reporting prevalence rates stratified by sex – found that a total of 64.6% of men and 89.5% of women visited a specialized physician in the past 12 months [34]. Comparisons have to be drawn very carefully as the DEGS1-sample was also comprised of people with a migration background and a weighting factor was applied to correct for deviations in the survey sample from the structure of the German population regarding migration background [21]. However, our results may hint to a lower utilization of specialist health care services among people with a migration background and may, therefore, be in line with other studies [25].

In the past decade, health policy efforts in Germany have been striving for an ambulatory health care in which general physicians act as gatekeepers for health care [11, 51]. The lack of associations of migration-sensitive factors with the utilization of specialized physicians may be related to the hypothesis that once patients have entered the health care system via a general practitioner, they are guided by these practitioners through the system. However, drawing conclusions on this issue is rather impossible as research is scarce on the role of general practitioners as gatekeepers and guides to specialized care for people with a migration background [25]. Our findings suggest that entry into the health care system may be affected by migration-sensitive factors as they were associated with lower utilization of general practitioners. This result may be explained by potential barriers to first access – usually via a general practitioner – into the health care system, for example, language barriers or lack of knowledge about the health care system [52]. In addition, discriminatory and racist experiences might explain utilization patterns. It is known from several studies that exposure to institutional racism in the health care setting and experiences of everyday discrimination in the receipt of health care results in lower utilization by people with a migration background [39, 41, 53].

### Factors associated with primary and specialist health care utilization

In our study, females had higher odds of having visited general practitioners and specialized physicians. Other studies substantiate this finding [14, 54, 55]. Besides reproductive differences in health care utilization (e.g., gynecologist visits [56]), more frequent health care utilization among females may be explained by gender-related socialization processes, for example, the development of a higher health awareness [14, 57].

People aged 65 years or above were more likely to access general practitioners and specialized physicians. Trends of higher health care utilization with increasing age have also been examined in other studies [14, 56] and are mostly due to increased prevalence of chronic diseases and combined health restrictions [58–60].

No association was observed between socioeconomic position and health care utilization. Also performing further analyses by testing alternatives (for example building categories above and below median, building categories based on quartiles) did not yield different results. This is in contrast to other studies which showed that people with a low socioeconomic position were more likely to contact general practitioners and less likely to contact specialized physicians compared to people with a higher socioeconomic position [14, 33, 34, 54]. However, in a systematic review of inequalities in health care utilization among migrants and non-migrants in Germany, findings suggested that factors related to migration background (such as language barriers or differences in need or information) determined utilization, despite socioeconomic differences. Socioeconomic position does not substantially clarify associations between migration background and health care utilization [17].

In accordance with previous studies, poorer self-rated health was associated with higher primary and specialized health care utilization. This result is in line with other research because the demand determines the utilization [14, 61].

The association between migration-sensitive factors and health care use is confirmed by other studies [16, 26]; therefore, a special emphasis should be put on these factors. Duration of residence in Germany for less than 2 years and a temporary residence status both decreased the odds of having visited a general practitioner. These characteristics occurred most frequently in the group with Syrian nationality. In recent years, persons with Syrian nationality have been among the group of asylum seekers with the highest immigration rates in Germany [62]. The nature and the benefits related to health care services are regulated in the Asylum-Seekers’ Benefits Act (“Asylbewerberleistungsgesetz”). In the survey period, the range of health care services available to asylum seekers was limited during the first 15 months of their stay in Germany, for example, to the treatment of acute illnesses [63]. The lower odds among persons with a temporary residence status may, therefore, be explained by restricted access to health care among the group of asylum seekers. These results highlight that the regulations imposed by the Asylum Seekers’ Benefits Act lead to inequalities in health care utilization and contribute to the reinforcement of social inequalities. Especially in times of a pandemic (such as COVID-19), limited access to health care services could have serious consequences for asylum seekers, a population group that is often exposed to poorer housing and working conditions (e.g., cramped housing, illegalized work in the informal sector) and poverty (e.g., no financial reserves) and, therefore, is already exposed to a higher risk of infection [64]. If infected, treatment initiation could be delayed, as asylum seekers may not see a general practitioner until they experience severe symptoms. At the same time, later detection of the disease could increase the risk of infecting more people in the surrounding community with the virus, for example, in collective accommodations for refugees.

Another possible explanation for lower odds of health services among persons residing in Germany for less than 2 years could be that they are unfamiliar with the structures of the German health care system. The importance and function of general practitioners in the country of origin and country of residence may differ a lot [18]. In Syria, for example, health care services are almost exclusively provided in health care centers and public hospitals [65]. Despite free access to health-related services, co-payments often have to be made that many people cannot afford. This often results in a postponement and an avoidance of health care utilization [65, 66]. Fears of upcoming costs in the country of residence could therefore discourage people from health care utilization.

### Differentiated consideration of migration-sensitive factors in health care research

We found associations between sociodemographic, health-related, and migration-sensitive factors and primary and specialist health care utilization among people with a migration background. These results suggest that a consideration of migration-sensitive characteristics, such as residence status or duration of residence, in health services research is helpful to gain detailed insights into the use of health care and potential barriers to its use among the heterogeneous group of people with a migration background. A focus in studies solely on a statistically defined migration background – an ascriptive category into which people are assigned – cannot capture associations regarding migration-sensitive related factors in a differentiated manner. For example, this category cannot provide any information about possible experiences of discrimination that may affect the use of health care services. A focus on these experiences when examining utilization would instead provide concrete approaches for the development and implementation of measures to improve access to health care. In summary, our results propose that health care research addressing people with a migration background should follow a diversity-sensitive approach by considering migration-sensitive factors alongside other diversity-sensitive factors, such as gender, education, religious affiliation, or sexual orientation [49, 67, 68].

### Limitations

Due to the sampling strategy and sample characteristics, our results might not be representative of all people with a migration background living in Germany. First, the sample was limited to five nationalities, which implies that the diversity of people with different nationalities living in Germany is not entirely represented in our study. Groups with other nationalities as well as those people with a migration background who have been naturalized were not considered and may have different utilization patterns to those observed. Additional research should investigate factors associated with primary and specialist health care utilization among people with a migration background on a representative level. Second, person-related data can only be deposited at the resident registration offices if the person has been registered at the registration authority of their municipality [44]. Specific groups of people with a migration background, who have not been registered, are therefore excluded from our study, for example people without legal residence status. Third, sample points in Brandenburg were drawn in cities with at least 33,000 inhabitants [69, 70]. As rural areas in Germany have a lower density of general practitioners and specialized physicians [11], the inclusion of more rural areas as sample points could possibly decrease the frequency of primary and specialist health care utilization. However, the proportion of people with a migration background among the general population in rural areas is smaller compared to urban areas. Fourth, more than three-quarters of the people with Syrian nationality had a temporary residence status, including a large proportion of asylum seekers. Access to health care and health care services are not standardized for asylum seekers in the federal states in Germany [63, 71]. The inclusion of other federal states could, therefore, have led to different results. Fifth, a recall bias may have affected the response behavior as the questions addressed events that may have taken place several months ago [10], whereas a socially desirable response behavior may not be assumed by questions about primary and specialist health care utilization [51]. Methodological biases cannot be completely precluded due to a different data distribution and low case numbers within some strata.

## Conclusions

This study delivers further insight into factors associated with primary and specialist health care utilization among people with a migration background as defined by nationality.

Differentiated analyses of migration-sensitive factors enabled us to identify that duration of residence in Germany and residence status affected the utilization of primary but not specialist health care services among the group of people with a migration background. Since sociodemographic (sex, age) and health-related (self-rated health) factors were associated with health care utilization, our findings highlight the importance of considering the heterogenous composition of the group of people with a migration background when investigating barriers to the use of primary and specialist health care services. In addition to our findings, further exploration of possible barrier mechanisms is relevant in order to reduce unequal health care access. More detailed information about potential barriers allows the development of specific strategies to improve access to health care services among the group of people with a migration background, which constitutes a large part of the total population in Germany.

Our study results cannot be representative of all people with a migration background living in Germany and thus, further research using a population-based representative sample is required to reveal patterns of health care utilization.

## Data Availability

The dataset analyzed during this study is available from the Robert Koch Institute upon reasonable request.

## Abbreviations

DEGS: German Health Interview and Examination Survey for Adults
EHIS: European Health Interview Survey
IMIRA: Improving Health Monitoring in Migrant Populations
RKI: Robert Koch Institute
SEP: socioeconomic position
